# Real-life clinical sensitivity of SARS-CoV-2 RT-PCR test in symptomatic patients

**DOI:** 10.1371/journal.pone.0251661

**Published:** 2021-05-21

**Authors:** Elisa Kortela, Vesa Kirjavainen, Maarit J. Ahava, Suvi T. Jokiranta, Anna But, Anna Lindahl, Anu E. Jääskeläinen, Annemarjut J. Jääskeläinen, Asko Järvinen, Pia Jokela, Hannimari Kallio-Kokko, Raisa Loginov, Laura Mannonen, Eeva Ruotsalainen, Tarja Sironen, Olli Vapalahti, Maija Lappalainen, Hanna-Riikka Kreivi, Hanna Jarva, Satu Kurkela, Eliisa Kekäläinen

**Affiliations:** 1 Division of Infectious Diseases, Inflammation Center, Helsinki University Hospital and University of Helsinki, Helsinki, Finland; 2 HUSLAB Clinical Microbiology, HUS Diagnostic Center, University of Helsinki and Helsinki University Hospital, Helsinki, Finland; 3 Translational Immunology Research Program and Department of Bacteriology and Immunology, University of Helsinki, Helsinki, Finland; 4 Biostatistics Consulting, Department of Public Health, University of Helsinki and Helsinki University Hospital, Helsinki, Finland; 5 Department of Respiratory Medicine, Heart and Lung Center, Helsinki University Hospital, University of Helsinki, Helsinki, Finland; 6 Department of Virology, Faculty of Medicine, University of Helsinki, Helsinki, Finland; 7 Department of Veterinary Biosciences, Faculty of Veterinary Medicine, University of Helsinki, Helsinki, Finland; Meyer Children’s University Hospital - University of Florence, ITALY

## Abstract

**Background:**

Understanding the false negative rates of SARS-CoV-2 RT-PCR testing is pivotal for the management of the COVID-19 pandemic and it has implications for patient management. Our aim was to determine the real-life clinical sensitivity of SARS-CoV-2 RT-PCR.

**Methods:**

This population-based retrospective study was conducted in March–April 2020 in the Helsinki Capital Region, Finland. Adults who were clinically suspected of SARS-CoV-2 infection and underwent SARS-CoV-2 RT-PCR testing, with sufficient data in their medical records for grading of clinical suspicion were eligible. In addition to examining the first RT-PCR test of repeat-tested individuals, we also used high clinical suspicion for COVID-19 as the reference standard for calculating the sensitivity of SARS-CoV-2 RT-PCR.

**Results:**

All 1,194 inpatients (mean [SD] age, 63.2 [18.3] years; 45.2% women) admitted to COVID-19 cohort wards during the study period were included. The outpatient cohort of 1,814 individuals (mean [SD] age, 45.4 [17.2] years; 69.1% women) was sampled from epidemiological line lists by systematic quasi-random sampling. The sensitivity (95% CI) for laboratory confirmed cases (repeat-tested patients) was 85.7% (81.5–89.1%) inpatients; 95.5% (92.2–97.5%) outpatients, 89.9% (88.2–92.1%) all. When also patients that were graded as high suspicion but never tested positive were included in the denominator, the sensitivity (95% CI) was: 67.5% (62.9–71.9%) inpatients; 34.9% (31.4–38.5%) outpatients; 47.3% (44.4–50.3%) all.

**Conclusions:**

The clinical sensitivity of SARS-CoV-2 RT-PCR testing was only moderate at best. The relatively high false negative rates of SARS-CoV-2 RT-PCR testing need to be accounted for in clinical decision making, epidemiological interpretations, and when using RT-PCR as a reference for other tests.

## Introduction

During the COVID-19 pandemic a central method for limiting the spread of SARS-CoV-2 has been the so-called “Test, Trace, Isolate” (TTI) approach promoted by the World Health Organization [1, 2]. A key feature of any laboratory test is its efficacy in detecting true positive cases. Evidence suggests a fair analytical sensitivity for the SARS-CoV-2 RT-PCR tests available on the market [3, 4]. However, reports suggest that clinically evident COVID-19 infections often go undetected by SARS-CoV-2 RT-PCR testing [5–9], as estimated by e.g. networked dynamic metapopulation models [6], and repeat-testing of patients [9].

A number of pivotal factors may decrease the overall sensitivity of testing and its usefulness in the TTI strategy. Preanalytical pitfalls such as suboptimal specimen collection may affect sample quality and hamper test sensitivity. Variation in viral shedding in different anatomical locations, and temporal variation in relation to disease onset can influence detection rates [10, 11].

High false negative rate complicates controlling the epidemic but it also has implications for healthcare settings [12]. Removal of infection control precautions in hospitalized patients due to a false negative test causes an occupational hazard for healthcare workers and can lead to nosocomial spread of the disease. Real-life sensitivity estimates in the initial reports [13, 14] were limited by small sample sizes and variable testing methods and reference standards.

We decided to evaluate the clinical sensitivity of SARS-CoV-2 RT-PCR in a population-based setting in the beginning of the epidemic with low level of transmission. To avoid bias created by the high pretest probability of inpatients, we included in our analysis also outpatients. We used manually curated clinical characteristics from a cohort of 3,008 individuals as the gold standard for the RT-PCR test. We focused on the sensitivity of the first SARS-CoV-2 RT-PCR test since for outpatients repeated testing is not often feasible. Our analysis that uses only non-dependent samples aims to avoid the bias created from repeated sampling of the same individuals. Our data can be directly used to inform the practicing clinicians and epidemiologists how well the RT-PCR performs in a low prevalence setting.

## Materials and methods

### Study design and participants

We present data from a retrospective study conducted from electronic, comprehensive medical records. The data were fully anonymized for the study. The study complies with the STARD reporting guidelines [15], and it was approved by the review board of the Helsinki University Hospital, Finland (HUS/157/2020-29), which waived the requirement for informed consent.

During the study period 4 March– 15 April 2020, 22,821 individuals underwent SARS-CoV-2 RT-PCR testing with a total of 1,938 test positive specimens at HUSLAB laboratory, Helsinki University Hospital, Finland, which serves the Helsinki Capital Area in Finland. S1 Fig shows the number of daily specimens and proportion of positive specimens at HUSLAB during the study period.

We reasoned that the pretest probability, i.e. the probability for testing positive, would be different for inpatients on the COVID-19 cohort wards and outpatients and decided to study these two populations separately.

#### Outpatient cohort

**Fig 1:**
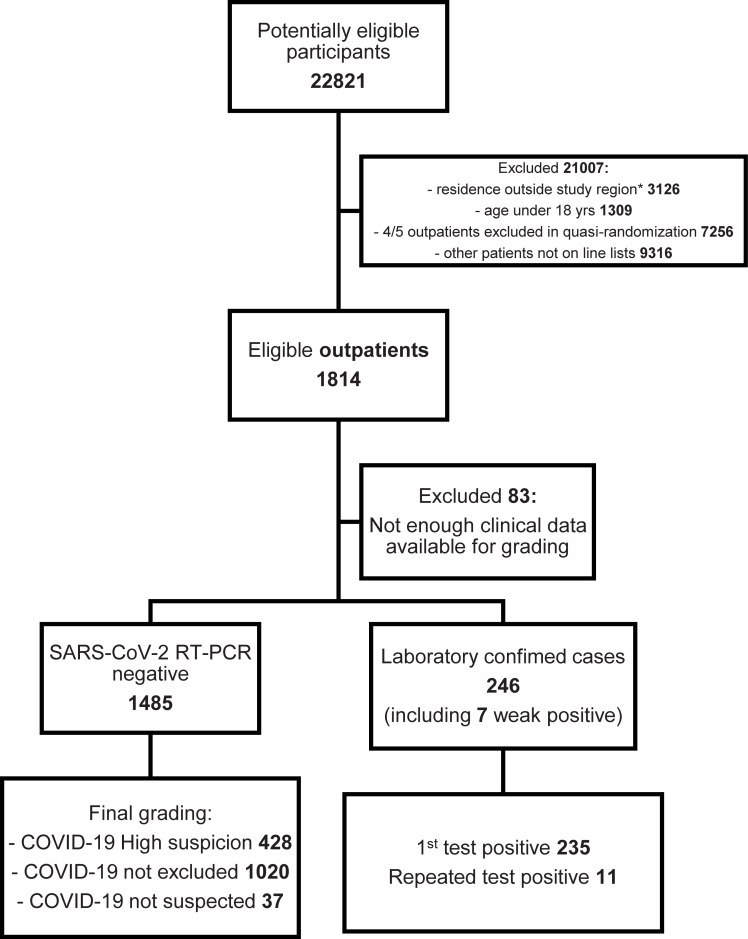
Selection of the outpatient cohort presented as a flowchart.

During the study period, the tested patients were generally symptomatic but the criteria which prompted testing varied slightly over time (S1 Table). Initially, persons returning from recognized epidemic areas and exhibiting respiratory symptoms within 14 days of return were primarily tested. The criteria were soon expanded to include symptomatic persons with risk factors, and all symptomatic healthcare workers. Outpatients fulfilling testing criteria were recorded manually with some clinical details on a line list. These lists were the most systematically collected dataset for the tested outpatients so we chose to sample our outpatient cohort from these lists. We performed systematic (quasi-random) sampling by including every fifth individual from the line lists. Along with practical advantages, this approach decreased the probability of sampling dependent individuals, such as members of the same family. Besides the clinical details on the line lists, we checked electronic medical records for comorbidities and other demographic details. Other exclusion criteria for outpatients were age below 18 years and residence outside of the Helsinki and Uusimaa Hospital District. Altogether 1,814 eligible outpatients (mean [SD] age, 45.4 [17.2] years; 69.1% women; 41.2% healthcare workers) were included in the study (Fig 1, Table 1).

**Table 1:** Patient demographic and clinical characteristics.

	Inpatients (n = 1,194)		Outpatients (n = 1,814)		Total (n = 3,008)	
	Number	%	Number	%	Number	%
Sex						
Male	654	54.8	560	30.9	1215	40.4
Female	540	45.2	1254	69.1	1794	59.6
Age, years						
Median (IQR)	66	51–77^a^	43	31–56^a^	51	36–69^a^
Mean (SD)	63.2	18.3	45.4	17.2	52.5	19.7
18–19	7		19		26	
20–29	58		350		408	
30–39	89		411		500	
40–49	128		355		483	
50–59	179		336		515	
60–69	213		130		343	
70–79	284		126		410	
80–79	173		72		245	
90–100	62		15		77	
100-	1		-		1	
Suspect grade for COVID-19 disease						
Not suspected	477	39.9	37	1.8	514	17.1
Not excluded	298	25.0	1020	58.1	1318	43.8
High suspicion	88	7.4	428	22.0	516	17.2
Laboratory confirmed	328	27.5	246	13.6	574	19.1
Not known	3	0.3	83	4.6	86	2.9
Other diagnosis confirmed						
Yes	554	46.4	31	1.7	585	19.4
No	600	50.3	1646	90.7	2246	74.7
Not known	40	3.4	137	7.6	177	5.9
ICU patients	158	13.2	-		158	5.3
Number of patients by sample count during the episode						
1 sample	1047	87.7	1558	85.9	2605	86.6
2 samples	104	8.7	210	11.6	314	10.4
3 samples	27	2.3	39	2.1	66	2.2
4 samples	12	1.0	7	0.4	19	0.6
5 samples	4	0.3	-		4	0.1
Total samples	1404		2123		3527	
Sample type (1^st^ sample)						
Nasopharyngeal+nasal	674+43	60.0	1015+37	58.0	1689+80	58.8
Oropharyngeal	132	11.1	297	16.4	429	14.3
Tracheal+bronchial+sputum	2+2+2	0.5	0+0+1	0.1	2+2+3	0.2
Sinus+lung biopsy	1+1	0.2	2+0	0.1	3+1	0.1
Not known	337	28.2	462	25.5	799	26.6
Healthcare worker						
Yes	59 ^b^	4.9	747 ^b^	41.2	806	26.8
No	1028	86.1	737	40.6	1765	58.7
Not known	107	9.0	330	18.2	437	14.5
Smoking						
Yes	191	16.0	139	7.7	330	11.0
Previous	268	22.4	154	8.5	422	14.0
No	439	36.8	438	24.1	877	29.2
Not known	296	24.8	1083	59.7	1379	45.8
Comorbidities ^c^						
Yes	794	66.5	367	20.2	1161	38.6
No	342	28.6	1152	63.5	1494	49.7
Not known	58	4.9	295	16.3	353	11.7
Pregnancy, if female						
Yes	11	2.0	27	2.2	38	2.1
No	480	88.9	500	39.9	980	54.6
Not known	49	9.1	727	58.0	776	43.3

^a^ Continuous variables reported as median and IQR (non-normally distributed variables) or mean and SD (normally distributed variables).

^b^ inpatients: 45/59 female (76,3%); outpatients: 633/747 female (84,7%).

^c^ heart disease, hypertension, lung disease, diabetes with organ damage, chronic kidney disease, dialysis, chronic liver disease, immunodeficiency, immunosuppressive medication or cancer.

#### Inpatient cohort

**Fig 2:**
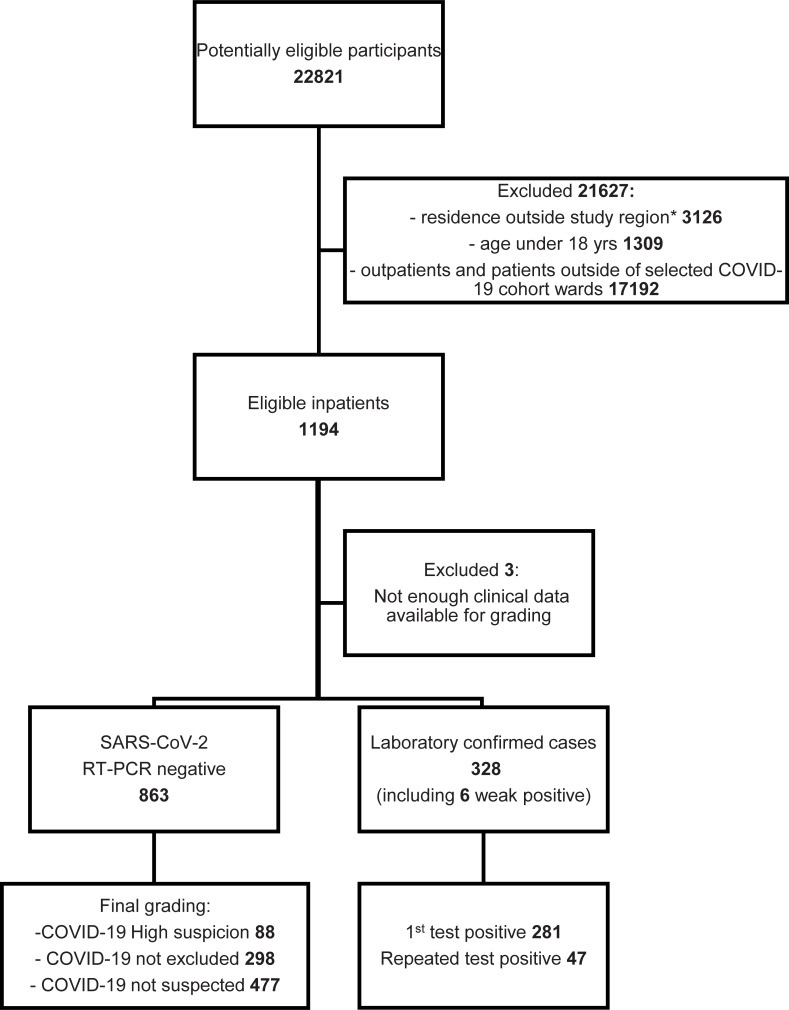
Selection of the inpatient cohort presented as a flowchart.

Patients with fever, respiratory or gastrointestinal symptoms, and/or difficulty in breathing were suspected for COVID-19 and treated in designated cohort wards: 11 wards and 6 ICUs in eight hospitals (list of wards in S2 Table). All patients aged >18 years admitted to one of the cohort wards were eligible for the study and only patients without a SARS-CoV-2 RT-PCR performed at the HUSLAB laboratory were excluded. These inpatients formed a consecutive case series of 1,194 individuals (mean [SD] age, 63.2 [18.3] years; 45.2% women) (Fig 2, Table 1).

Altogether 12.3% (147/1194) of inpatients and 14.1% (256/1814) of outpatients were sampled for SARS-CoV-2 RT-PCR testing more than once during a specific disease episode (Table 1). The descriptive statistics of the tested individuals and subgroups are presented in Tables 1 and 2 and S2 Fig.

**Table 2:** Patient demographic and clinical characteristics by clinical suspicion of COVID-19 in inpatients (A) and outpatients (B). Three patients in the inpatient cohort could not be classified. ‘Not suspected’: deemed by the clinician as non-compatible with COVID-19 disease or were diagnosed with another acute disease; ‘Not excluded’: no other diagnosis recorded explaining their current symptoms, and COVID-19 disease could not be excluded. ‘High suspicion’: physician in charge of the treatment recorded the suspicion on clinical grounds to the electronic patient record, OR the patient fulfilled a set of pre-defined clinical and exposure criteria (see Methods); ‘Laboratory confirmed’: tested positive from the first sample taken or with repeated testing with SARS-CoV-2 RT-PCR.

**A**									
	**COVID-19 No Suspicion (n = 477)**		**COVID-19 Not Excluded (n = 298)**		**COVID-19 High Suspicion (n = 88)**		**COVID-19 Laboratory Confirmed (n = 328)**		P Value COVID-19 High Suspicion vs. Covid lab.confirmed
	**Number**	**%**	**Number**	**%**	**Number**	**%**	**Number**	**%**	
Sex									.16^a^
Male	255	53.5	172	57.7	41	46.6	183	55.8	
Female	222	46.5	126	42.3	47	53.4	145	44.2	
Age, years									
Median (IQR)^b^	71	56–80	69	52–78	55.5	41–70.25	58	47.75–72	
Range	18–103		18–96		18–96				
Other diagnosis confirmed									
Yes	448	93.9	92	30.9	ND		ND		
No	17	3.6	184	61.7	ND		ND		
Not known	12	2.5	22	7.4	ND		ND		
Influenza A, Influenza B, and RSV									
Total tested	395	82.8	261	87.6	79	89.8	186	56.7	< .001^a^
Influenza A positive	6	1.3	0	0	0		0		NA
Influenza B positive	4	0.8	0	0	0		0		NA
RSV positive	24	3.1	9	3.0	0		0		NA
Other virus finding									
Total tested	15	3.1	20	6.7	7	8.0	1	0.3	< .001^a^
Coronavirus 229E/NL63/OC43	2				-		-		NA
Rhinovirus	-		1		-		-		NA
Rhinovirus and Coronavirus 229E/NL63/OC43	1				-		-		NA
ICU patients	44	9.2	22	7.4	7	7.8	82	25.0	< .001^a^
Time between symptom onset and first SARS-CoV-2 RT-PCR test									
<1 day	152	31.9	71	23.8	7	8.0	22	6.7	
1–2 days	112	23.5	59	19.8	12	13.6	55	16.8	
3–4 days	46	9.6	39	13.1	15	17.0	50	15.2	
5–6 days	29	6.1	28	9.4	7	8.0	58	17.7	
7–14 days	61	12.8	54	18.1	38	43.2	132	40.2	
>14 days	38	8.0	31	10.4	7	8.0	8	2.4	
No data	39	8.2	16	5.4	2	2.3	3	0.9	
Fever									< .001^c^
Yes	233	48.8	193	64.8	67	76.1	300	91.5	
No	187	39.2	85	28.5	20	22.7	21	6.4	
Not known	57	11.9	20	6.7	1	1.1	7	2.1	
Upper respiratory symptoms									.86^a^
Yes	119	24.9	83	27.9	38	43.2	131	39.9	
No	218	45.7	109	36.6	23	26.1	90	27.4	
Not known	140	29.4	106	35.6	27	30.7	107	32.6	
Lower respiratory symptoms									.52^c^
Yes	282	59.1	240	80.5	84	95.5	299	91.2	
No	134	28.1	39	13.1	2	2.3	14	4.3	
Not known	61	12.8	19	6.4	2	2.3	15	4.6	
Infectious finding in chest X-ray/CT									.33^c^
Yes	78	16.4	103	34.6	72	81.8	266	81.1	
No	337	70.6	181	60.7	12	13.6	55	16.8	
Not done	28	5.9	9	3.0	2	2.3	2	0.6	
Not known	34	7.1	5	1.7	2	2.3	5	1.5	
Altered sense of smell									.24^a^
Yes	3	0.6	2	0.7	3	3.4	27	8.2	
No	59	12.4	24	8.1	13	14.8	38	11.6	
Not known	415	87.0	272	91.3	72	81.8	263	80.2	
Gastrointestinal symptoms									.03^a^
Yes	137	28.7	65	21.8	32	36.4	155	47.3	
No	146	30.6	109	36.6	21	23.9	90	27.4	
Not known	194	40.7	124	41.6	35	39.8	83	25.3	
Thrombosis									.26^c^
Yes	18	3.8	9	3.0	6	6.8	10	3.0	
No	261	54.7	208	69.8	56	63.6	220	67.1	
Not known	198	41.5	81	27.2	26	29.5	98	29.9	
Contact in 14 days to symptomatic COVID-19									.03^a^
Yes	15	3.1	10	3.4	18	20.5	117	35.7	
No	214	44.9	124	41.6	33	37.5	101	30.8	
Not known	248	52.0	164	55.0	37	42.0	110	33.5	
Travel in 14 days									.11^a^
To defined risk areas ^d^	7	1.5	3	1.0	4	4.5	28	8.5	
Other	11	2.3	12	4.0	10	11.4	19	5.8	
No travel abroad	300	62.9	208	69.8	55	62.5	227	69.2	
Not known	159	33.3	75	25.2	19	21.6	54	16.5	
Healthcare worker:									.13^c^
Yes	10	2.1	8	2.7	5	5.7	36	11.0	
No	432	90.6	269	90.3	68	77.3	256	78.0	
Not known	35	7.3	21	7.0	15	17.0	36	11.0	
Clinical severity grade									.23^a^
Low	ND		ND		-		2	0.6	
Medium	ND		ND		62	70.5	198	60.4	
Severe ^e^	ND		ND		23	26.1	120	36.6	
Not known	ND		ND		3	3.4	8	2.4	
Smoking									.001^a^
Yes	106	22.2	57	19.1	13	14.8	14	4.3	
Quitted	95	19.9	87	29.2	19	21.6	66	20.1	
No	137	28.7	88	29.5	37	42.0	177	54.0	
Not known	139	29.1	66	22.1	19	21.6	71	21.6	
Comorbidities									.19^c^
Yes	364	76.3	230	77.2	49	54.4	150	45.7	
No	80	16.8	66	22.1	39	43.3	158	48.2	
Not known	33	6.9	2	0.7	2	2.2	20	6.1	
B									
	**COVID-19 No Suspicion (n = 37)**		**COVID-19 Not Excluded (n = 1020)**		**COVID-19 High Suspicion (n = 428)**		**COVID-19 Laboratory Confirmed (n = 246)**		**P Value COVID-19 High Suspicion vs. Covid lab.confirmed**
	**Number**	**%**	**Number**	**%**	**Number**	**%**	**Number**	**%**	
Sex									.004^a^
Male	17	45.9	256	25.1	151	35.3	115	46.7	
Female	20	54.1	764	74.9	277	64.7	131	53.3	
Age, years									
Median (IQR)^b^	61	34–76	45	32–58	40	30–51	42	31–55	
Range	18–89		18–98		18–91		18–94		
Other diagnosis confirmed									.13^c^
Yes	29	78.4	-		-		-		
No	7	18.9	957	93.8	417	97.4	234	95.1	
Not known	1	2.7	63	6.2	11	2.6	12	4.9	
Influenza A, Influenza B and RSV									
Total tested	21	56.8	104	10.2	58	13.6	17	6.9	.01^a^
Influenza A positive	3		0		0		0		NA
Influenza B positive	4		0		0		0		NA
RSV positive	2		0		0		0		NA
Other virus finding									
Total tested	4		2		3		1		>.99^c^
Rhinovirus	1		-		-		-		NA
Time between symptom onset and first SARS-CoV-2 RT-PCR test									
<1 day	5	13.5	28	2.7	14	3.3	15	6.1	
1–2 days	12	32.4	260	25.5	120	28.0	74	30.1	
3–4 days	4	10.8	203	19.9	84	19.6	52	21.1	
5–6 days	7	18.9	117	11.5	48	11.2	30	12.2	
7–14 days	3	8.1	246	24.1	98	22.9	40	16.3	
>14 days	1	2.7	65	6.4	19	4.4	5	2.0	
No data	5	13.5	101	9.9	45	10.5	30	12.2	
Fever									< .001^a^
Yes	11	29.7	122	12.0	55	12.9	90	36.6	
No	16	43.2	337	33.0	144	33.6	59	24.0	
Not known	10	27.0	561	55.0	229	53.5	97	39.4	
Upper respiratory symptoms									.81^c^
Yes	10	27.0	371	36.4	179	41.8	102	41.5	
No	7	18.9	15	1.5	6	1.4	5	2.0	
Not known	20	54.1	634	62.2	243	56.8	139	56.5	
Lower respiratory symptoms									.51^a^
Yes	19	54.3	440	43.1	198	46.3	123	50.0	
No	4	10.8	29	2.8	11	2.6	8	3.3	
Not known	14	37.8	551	54.0	219	51.2	115	46.7	
Infectious finding in chest X-ray/CT									.18^c^
Yes	1	2.7	4	0.4	4	0.9	5	2.0	
No	14	37.8	57	5.6	9	2.1	11	4.5	
Not done	16	43.2	613	61.0	244	57.0	130	52.8	
Not known	6	16.2	346	33.9	171	40.0	100	40.7	
Altered sense of smell									< .001^c^
Yes	1	2.7	5	0.5	8	1.9	23	9.3	
No	1	2.7	2	0.2	-		1	0.4	
Not known	35	94.6	1013	99.3	420	98.1	222	90.2	
Gastrointestinal symptoms									.19^c^
Yes	2	5.4	55	5.4	15	3.5	14	5.7	
No	5	13.5	9	0.9	1	0.2	2	0.8	
Not known	30	81.1	956	93.7	412	96.3	230	93.5	
Thrombosis									.49
Yes	-		-		-		-		
No	17	45.9	117	11.5	36	8.4	25	10.2	
Not known	20	54.1	903	88.5	392	91.6	221	89.8	
Contact in 14 days to symptomatic COVID-19									< .001^a^
Yes	7	18.9	3	0.3	299	69.9	141	57.3	
No	12	32.4	341	33.4	32	7.5	16	6.5	
Not known	18	48.6	676	66.3	97	22.4	89	36.2	
Travel in 14 days									.005^a^
To defined risk areas ^d^	3	8.1	12	1.2	131	30.6	49	19.9	
Other	-		197	19.3	16	3.7	16	6.5	
No travel abroad	24	64.9	626	61.4	205	47.9	121	49.2	
Not known	10	27.0	185	18.1	76	17.8	60	24.4	
Healthcare worker:									< .001^a^
Yes	6	16.2	533	52.3	118	27.6	59	24.0	
No	28	75.7	398	39.0	170	39.7	127	51.6	
Not known	3	8.1	89	8.7	140	32.9	60	24.4	
Clinical severity grade:									.001^c^
Low	ND		ND		423	98.8	229	93.1	
Medium	ND		ND		2	0.5	2	0.8	
Severe	ND		ND		-		-		
Not known	ND		ND		3	0.7	15	6.1	
Smoking:									.80^c^
Yes	3	8.1	92	9.0	29	6.8	12	4.9	
Quitted	8	21.6	107	10.5	24	5.6	14	5.7	
No	9	24.3	270	26.5	95	22.2	55	22.4	
Not known	17	45.9	551	54.0	280	65.4	165	67.1	
Comorbidities:									>.99^a^
Yes	19	5.4	248	24.3	56	13.1	33	13.4	
No	15	40.5	653	64.0	285	66.6	163	66.3	
Not known	3	8.1	119	11.7	87	20.3	50	20.3	

^a^ Chi-squared test.

^b^ Continuous variables reported as median (IQR).

^c^ Fisher exact test.

^d^ defined risk areas were Austria Tiroli, Northern Italy, Spain, China and South Korea.

^e^ admitted to ICU OR recorded respiratory rate of ≥30/min OR oxygen saturation ≤85% (with or without supplemental oxygen at any stage).

ND = not determined.

NA = not applicable.

### Index testing

SARS-CoV-2 RT-PCR testing was conducted by one of the following methods (gene targets): Cobas® SARS-CoV-2 test kit on the CobasC® 6800 system (*orf1ab* and *E*) (Roche Diagnostics, Basel, Switzerland), Amplidiag® COVID-19 test (*orf1ab* and *N*) (Mobidiag, Espoo, Finland,) and a laboratory-developed test based on a protocol recommended by WHO (*N*) (non-exponential amplification curves and amplification with cycle treshold values >34 in the laboratory-developed test were reanalysed with either Cobas or Amplidiag) [16]. The specifics and analytical performance of these methods in our laboratory setting have been described previously [4]. Samples were collected with nasopharyngeal swabs (FLOQSwab, Copan, Brescia, Italy) (proportion of nasopharyngeal samples: 60% inpatients, 58% outpatients) but oropharyngeal swabs were used in a proportion of patients (11.1% inpatients, 16.4% outpatients) due to global shortage of nasopharyngeal swabs. Other specimens types (0.7% inpatients, 0.2% outpatients) were tracheal, brochial, and sputum specimens, as well as sinus and lung biopsies The specimen type was unknown in 28.2% of inpatients and 25.5% of outpatients.

Samples were analysed in median 24 hours after collection. As per our laboratory’s standard operating procedure, samples with failed results were reanalysed and only qualified results were included. 13 weak positive results which became positive in >35 PCR cycles (7 outpatients and 6 inpatients) were included.

### Reference standard used in the study

Since no gold standard for COVID-19 diagnosis exists, we decided to use high clinical suspicion for COVID-19 as the reference standard for the RT-PCR test. We systemically graded the clinical suspicion for COVID-19 based on a combination of symptoms, clinical findings, and recorded exposure to laboratory confirmed COVID-19 cases or travel history to epidemic areas. The criteria were based on CDC’s and ECDC’s case definitions for COVID-19 in April 2020. Electronic patient records or line lists were reviewed by a team consisting of senior residents in Infectious Diseases and Clinical Microbiology, medical students, and research nurses. Patients’ medical history, symptoms, and epidemiological information were collected into a Microsoft Access® database according to the pre-defined criteria. Chest X-ray and CT findings indicating chest infection were recorded according to radiologist’s interpretation. The team collecting the data were aware of the SARS-CoV-2 RT-PCR test result when collecting the data.

The clinical suspicion for COVID-19 disease was graded as follows:

1. ‘Not suspected’ patients were deemed by the clinician as non-compatible with COVID-19 disease or were diagnosed with another acute disease.
2. ‘Not excluded’ patients had no other diagnosis recorded explaining their current symptoms, and COVID-19 disease could not be excluded.
3. ‘High suspicion’ patients were considered to suffer from a probable COVID-19 if the physician in charge of the treatment recorded the suspicion on clinical grounds to the electronic patient record, OR the patient fulfilled at least one of the following criteria:
  - respiratory symptoms and/or fever and/or diagnostic finding for infection in chest X-ray/CT and travel history to epidemic regions at the time of the study i.e. Tirol/Austria, Northern Italy, Spain, Iran, South Korea, or China during the preceding 14 days.
  - respiratory symptoms and fever and diagnostic finding in chest X-ray/CT during April 2020 (time criterion based on the changed epidemiological situation).
  - respiratory or gastrointestinal symptoms or fever or diagnostic finding in chest X-ray/CT and a close contact with a laboratory confirmed COVID-19 patient during the preceding 14 days prior to disease onset.
4. ‘Laboratory confirmed’ patients (regardless of their clinical presentation) were those individuals that tested positive for SARS-CoV-2 RT-PCR during the study period. At the time of the study, only symptomatic individuals were tested, so the laboratory confirmed group does not include any asymptomatic cases.

### Sample size calculation

We estimated the minimum sample size needed for outpatients based on Bujang et al. [17], with a minimal statistical power of 80% and type I error <0.05. Sample size calculation for sensitivity requires a prevalence estimation in the target population. During the study period, the median positivity rate was 9.6% (S1 Fig) so we estimated a 10% prevalence for the tested population. Published estimates from small cohorts [18, 19] available at the time reported sensitivity of the SARS-CoV-2 RT-PCR to be on average 70%. Based on these estimates the minimum sample size of outpatients for null hypothesis of sensitivity of 70% was 1,550. We performed another sample size calculation by using the nomogram described by Carley et al. [20] which accounts for confidence intervals (CI): with CI of 93% and prevalence 10%, 70% sensitivity would require a minimum sample size of 1,600.

### Group comparisons

To detect if the high suspicion and the laboratory confirmed groups were comparable and if there would be significant confounding factors between the groups that were used for sensitivity calculations, we compared demographic and clinical characteristics between them (Tables 1 and 2). To compare these two groups with respect to the categorical variables, we used the Pearson’s Chi-squared test without or with Yate’s correction for continuity or the Fisher’s exact test, as appropriate. For the extensive contingency tables with the excess of small (expected) frequencies, we assessed the simulated p-value of the Fisher’s test based on 20,000 replicates. The differences in the age distribution were assessed using the Mann-Whitney U-test. These comparisons were performed separately within the inpatients and outpatients.

### Analysis of sensitivity

Two approaches were deployed to calculate SARS-CoV-2 RT-PCR sensitivity: repeat-tested laboratory confirmed patients, and patients with a high clinical suspicion of COVID-19.

For the laboratory confirmed patients, the sensitivity values were calculated based on the first RT-PCR test of each patient. All patients who tested RT-PCR positive during a specific disease episode were considered laboratory confirmed. Of these, the first samples with a negative RT-PCR test result were considered false negatives, while the first samples with a positive result were considered true positives. The same disease episode would include samples taken ≤14 days apart.

For the high clinical suspicion group, the sensitivity was calculated by considering those patients that were graded as high suspicion but never tested positive as false negative cases.

The 95% CIs for (binomial) sensitivity were calculated by using the Wilson-Score method, which is based on inverting the z-test for a single proportion and provides more reliable coverage than the alternatives. We performed comparisons of sensitivity between the subgroups by using the independent sample tests for binomial proportions, including Chi-squared test without or with Yate’s correction for continuity or the Fisher’s exact test, as appropriate. For the extensive contingency tables with the excess of small (expected) frequencies, we assessed the simulated p-value of the Fisher’s test based on 20,000 replicates. We set the confidence level at 5%. All calculations were performed using the R software.

## Results

### Demographics of the study population and clinical comparison between study groups

In all, 3,008 individuals were eligible for this study (Figs 1 and 2): 1,814 outpatients and 1,194 inpatients. Altogether 83 eligible outpatients (4.6%) and 3 inpatients (0.3%) were excluded from the final analysis due to insufficient data for the grading of clinical suspicion.

The inpatients were on average older than outpatients, comorbidities were more common, and the male sex was slightly overrepresented (Table 1). Healthcare workers and women were overrepresented in the outpatient population reflecting the distribution of the whole tested population, as reported before [21].

All patients were categorized by a clinical grade of suspicion (not suspected / not excluded / high suspicion / laboratory confirmed) for COVID-19 based on criteria described in Methods (Table 1). To detect if our grading created systematic bias or if there were significant confounding factors present between the groups, we compared test negative patients that were deemed as high suspicion to laboratory confirmed patients (Table 2). There were no significant differences in sex or age distribution between these groups, but patients treated in the intensive care unit were overrepresented in the laboratory confirmed hospitalized patients (Table 2A). Laboratory confirmed patients were also more often febrile and had had contact with laboratory confirmed symptomatic COVID-19 cases (Table 2A and 2B). Laboratory confirmed patients also had more often gastrointestinal symptoms than patients in the high suspicion group (Table 2A and 2B).

In the outpatients, the high suspicion group had a higher proportion of females and healthcare workers compared to laboratory confirmed cases. This was expected based on the overall higher testing rate of both [21]. Again, the laboratory confirmed cases were more often febrile. Since our grading criteria included exposure to symptomatic COVID-19 patients or travel to epidemic areas, these factors were more common in the high suspicion group that tested negative, than in the laboratory confirmed group (Table 2B).

### Sensitivity of the first SARS-CoV-2 RT-PCR in inpatients and outpatients

The sensitivity of SARS-CoV-2 RT-PCR was calculated with two different denominators (Table 3). We first calculated the sensitivity with laboratory confirmed cases, i.e. repeat-tested patients as a denominator, yielding the highest sensitivity estimates in this study, as follows: 85.7% for inpatients; 95.5% for outpatients, and 89.9% for all. Due to low number of repeat-tested patients (N = 11), the calculation for outpatients here is unreliable.

**Table 3:** Sensitivity calculations for the first SARS-CoV-2 RT-PCR test.

Denominator		Inpatients	Outpatients	All
COVID-19 Laboratory confirmed patients	N/N	281/328	235/246	516/574
	**Sensitivity (%)**	**85.7%**	**95.5%**	**89.9%**
	95% CI	(81.5–89.1%)	(92.2–97.5%)	(88.2–92.1%)
COVID-19 Laboratory confirmed + High suspicion patients	N/N	281/416	235/674	516/1090
	**Sensitivity (%)**	**67.5%**	**34.9%**	**47.3%**
	95% CI	(62.9–71.9%)	(31.4–38.5%)	(44.4–50.3%)

The numerator for all calculations is the number of patients that tested positive with first sample.

The sensitivity was then calculated by including in the denominator patients that were graded as high suspicion but never tested positive (from one or more tests conducted within the study period), yielding the following sensitivity values: 67.5% for inpatients, 34.9% for outpatients and 47.3% for all. Thus, the lowest calculated sensitivity estimate in this study was for outpatients with high suspicion.

The delay between disease onset and testing was longer for inpatients than outpatients (Table 2). We could not detect a significant difference in the delay to first test between the laboratory confirmed cases and the high suspicion group in either cohort (S3 Fig, Fisher’s Exact Test p = 1 when “No data” category excluded). However, for inpatients, information on the delay was missing more often in the high suspicion group (2.3%) compared to the laboratory confirmed (0.9%, p = 0.026) (Table 2). For outpatients, information on the delay was missing less often in the high suspicion group (10.5%) as compared to the laboratory confirmed (12.2% p = 0.026) (Table 2).

We could not detect a significant difference between the sensitivity of nasopharyngeal and oropharyngeal samples in the inpatients (p = 0.51, Chi-squared test), outpatients (p = 0.22) or all patients (p = 0.66) (S3 Table; S4 Fig). However, data on the specimen type was missing in 20.4% (inpatients) and 17.4% (outpatients) of the cases.

### Delay between symptom onset and positive test result

**Fig 3:**
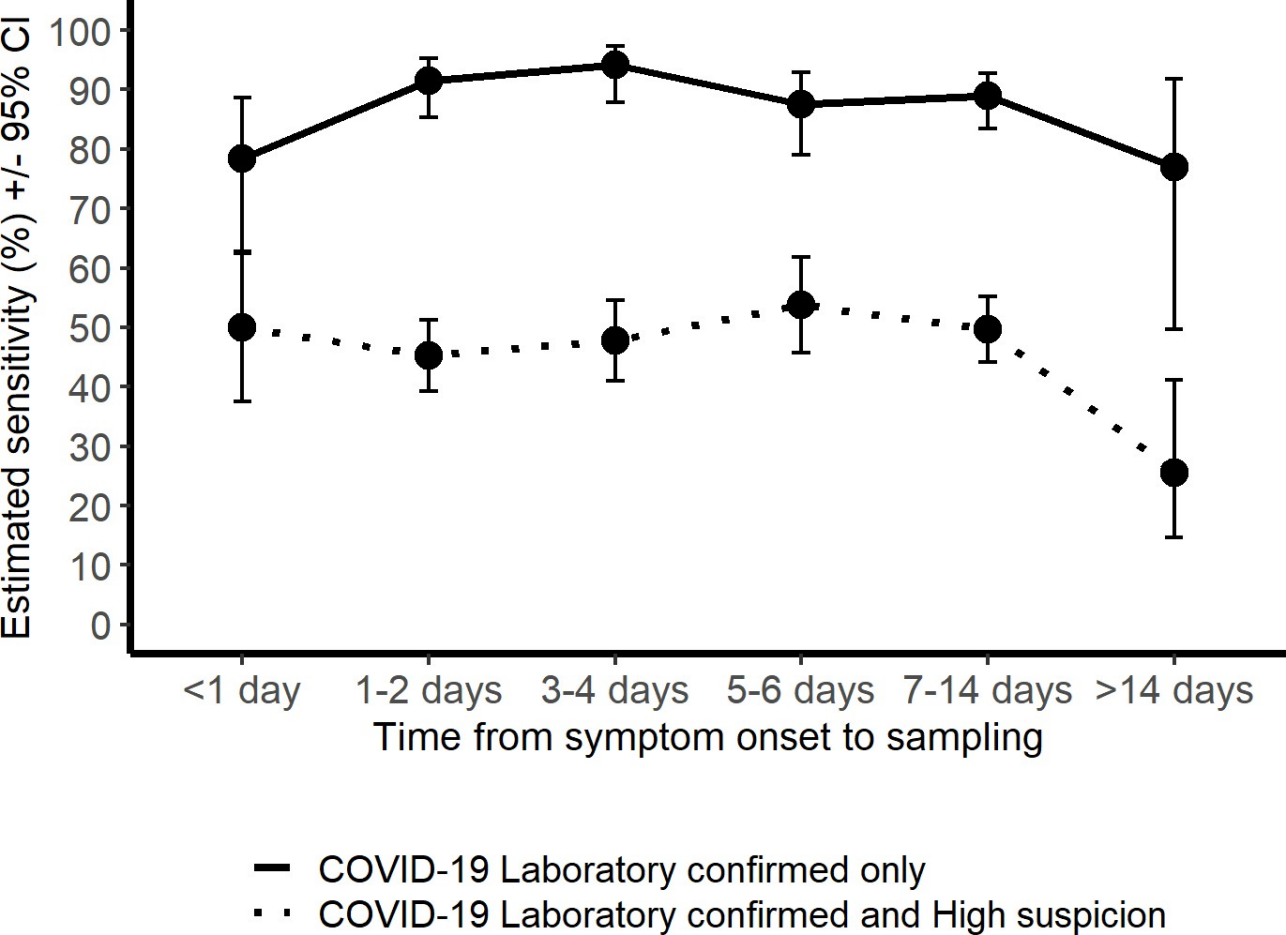
Clinical SARS-CoV-2 RT-PCR sensitivity estimates in the laboratory confirmed, and in the laboratory confirmed and high suspicion group combined, presented according to delay (days) from symptom onset to sampling.

To estimate the delay from disease onset for highest clinical sensitivity for SARS-CoV-2 RT-PCR, we calculated sensitivities for different time frames. To achieve reliable group sizes, both cohorts were pooled together. There was no significant difference in the test sensitivity according to delay from onset, calculated for the laboratory confirmed cases alone, and with the high clinical suspicion group included (P = 0.1013 Fisher’s Exact Test for Count Data with simulated p-value, based on 20000 replicates; Fig 3). Detailed sensitivity calculations per delay from disease onset are presented in S4 Table.

## Discussion

Wide-spread testing and contact tracing together with social distancing has been promoted as the tool that prevents new lockdowns–without clear understanding of how well the SARS-CoV-2 RT-PCR test performs. Here, we used clinical suspicion as the gold standard to estimate the clinical sensitivity of the test. We also included a sensitivity calculation based on the repeat-tested individuals where RT-PCR acts as the gold standard for itself.

A previous large-scale sensitivity estimate for SARS-CoV-2 molecular testing was based only on repeat-tested individuals [9, 22]. This approach overestimates the sensitivity. Repeated testing is done mostly on inpatients who have a strong clinical suspicion, rendering high pre-test probability. We sought to overcome this limitation by including a large cohort of outpatients. From an epidemiological point-of-view, understanding the clinical sensitivity for mild cases is important. An RT-PCR sensitivity of 64% for exposed family members systemically tested with serology was recently reported [23]. This is in line with our sensitivity estimation for inpatients. Another small study found an 86.2% RT-PCR sensitivity in symptomatic COVID-19 patients in comparison with convalescent antibody [24]. Interestingly, a recent Cochrane meta-analysis on thoracic imaging of COVID-19 patients found an 87.9% pooled sensitivity for chest CT and 80.6% for chest X-ray [25]. However, diagnostic imaging is mainly performed on inpatients that can overestimate the sensitivity. Our analysis was done in a low prevalence setting. Thus, the negative predictive value for the RT-PCR test was high (89%) for the outpatients even though the clinical sensitivity was low (35%), assuming all COVID-19 excluded cases were true negatives. High false negative rates reduce the negative predictive value of testing. This is particularly problematic when the prevalence of the disease increases. In such settings, it will impair effective use of wide-spread testing.

For health-care facilities the message of our data is different: a single negative result cannot be trusted to rule out COVID-19 in patients with suitable symptoms. Our data show that the sensitivity of the repeat-tested inpatients was high (86%), and in line with previous reports on repeated testing [9, 22]. When the sensitivity of the COVID-19 PCR test was judged based on the laboratory confirmed and high clinical suspicion patients the estimated sensitivity of the test dropped to around 68%. Our results emphasize the importance of repeated sampling but it also highlights the importance to evaluate the patient’s clinical presentation carefully.

This study estimated test sensitivity both with repeat-tested patients and by using clinical suspicion as a gold standard. The estimated sensitivity (89.9%) for repeat-tested patients is probably an overestimation: samples from a single individual are not independent and there is often a clear clinical rationale, i.e. high clinical suspicion for COVID-19 that necessitates the repeated testing, leading to increased pretest probability. In addition, hospitalized patients often present with a more severe disease with higher viral loads and longer viral nucleic acid shedding than individuals with mild symptoms [26], again leading to overestimation of test sensitivity. Our data shows that laboratory confirmed cases in both outpatients and hospitalized patients were more often febrile than the high clinical suspicion cases even though almost all other symptoms were comparable between the groups. This could indicate that the more severe cases were more often detected with RT-PCR. In contrast, the estimate which included both laboratory confirmed and high suspicion outpatients (34.9%), is likely an underestimation as COVID-19 symptoms are shared with other respiratory infections. In all, we conclude that the group which included both laboratory confirmed and high suspicion inpatients likely yielded the most realistic sensitivity estimate (67.5%).

Generally the first symptomatic days are considered best for virus detection from the upper airways [27, 28]. Due to the limited sample size our analysis could not detect a definitive time-point for highest sensitivity. However, even with this under-powered estimation, we should have detected major trends.

The study had several limitations. All patients were considered symptomatic so the estimates cannot be generalized to asymptomatic patients. The clinical criteria were set based on the information available in April 2020. While the core symptoms have remained the same, understanding of COVID-19 presentations has since increased. The clinical diagnosis of COVID-19 is notoriously hard as the symptoms are variable and overlap with many other similar conditions. In the hospitalized patients the testing coverage for other viral pathogens was extensive, and circulation of influenza and RSV was very limited at the time. However, most outpatients in this study were not tested for other potential viral pathogens. Potential information bias was introduced by the sometimes undetailed clinical records of outpatients. Reporting bias for more detailed symptoms most likely exists, especially for the laboratory confirmed outpatient cases. The specimen types recorded in the sample referrals may have contained errors. While pre-defined clinical criteria were used for grading, the data were collected retrospectively and the data collectors were aware of the index test result.

Large scale molecular testing has permanently changed the practice of clinical microbiology. RT-PCR for SARS-CoV-2 detection has many limitations as a labor intensive test with a relatively slow throughput. This has led to unbearable delays in results. Multiple solutions are being developed: point-of-care viral antigen detection [29], sample pooling [30], and self-sampling [31]. All these approaches, which use RT-PCR as a reference, quite consistently report lower sensitivity than RT-PCR. It is thus evident that all our current testing options are far from optimal in detecting all COVID-19 cases. In controlling of the ongoing pandemic, we need focused research to find an appropriate balance in the tradeoff between test sensitivity, and speed and ease of testing in each epidemiological setting.

## Supporting information

S1 Fig Number of SARS-CoV-2 RT-PCR tests conducted at the HUSLAB laboratory, and positivity rate over the study period 4 March– 15 April 2020. Median and mean positivity rates were 9.6% and 10%, respectively.
(TIF)

S2 Fig Age distribution of the study population as histograms. A. Inpatients. B. Outpatients.
(TIFF)

S3 Fig Delay to first SARS-CoV2 RT-PCR test (days) in the laboratory confirmed cases and the high suspicion group. A. Inpatients B. Outpatients.
(TIFF)

S4 Fig Visualisation of clinical sensitivity estimates for inpatients and outpatients according to specimen type. (TIFF)

S1 Table List of SARS-CoV-2 RT-PCR testing criteria over the study period. (DOCX)

S2 Table List of COVID-19 cohort wards. Inpatients were admitted to these COVID-19 cohort wards during the study period. Both laboratory confirmed and suspected COVID-19 patients were admitted to the cohort wards, except for two wards (COV_KNKINF and COV_KITEHO) to which only laboratory confirmed cases were admitted.
(DOCX)

S3 Table Estimated SARS-CoV-2 RT-PCR sensitivity values in the laboratory confirmed and high suspicion group combined according to specimen type in the first SARS-CoV-2 RT-PCR test. (DOCX)

S4 Table Estimated SARS-CoV-2 RT-PCR sensitivity values in the laboratory confirmed, and in the laboratory confirmed and high suspicion group combined according to delay (days) from symptom onset to first SARS-CoV-2 RT-PCR test. (DOCX)
