# Supplementary figures and images for: Real-life clinical sensitivity of SARS-CoV-2 RT-PCR test in symptomatic patients
Source: PLoS One. 2021 May 21;16(5):e0251661. doi: 10.1371/journal.pone.0251661 (PMC8139477; doi:10.1371/journal.pone.0251661)

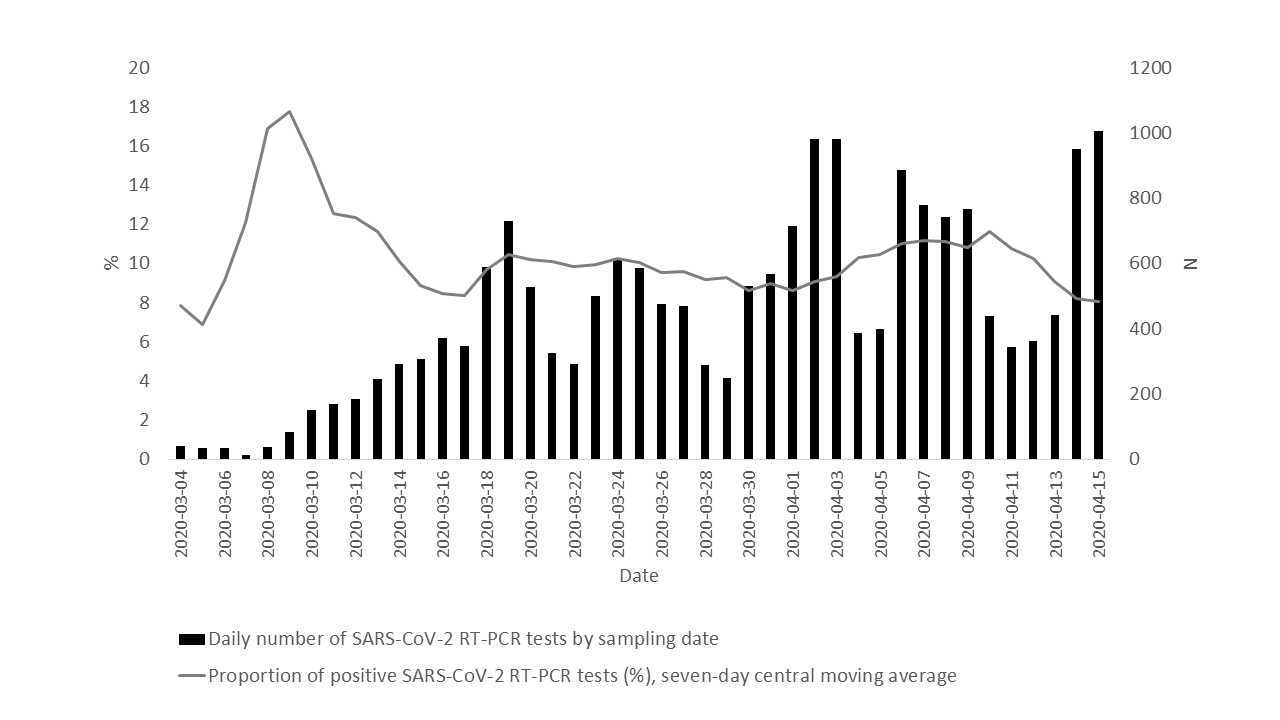

Supplement: S1 Fig — Median and mean positivity rates were 9.6% and 10%, respectively. (TIF) [file pone.0251661.s001.tif]

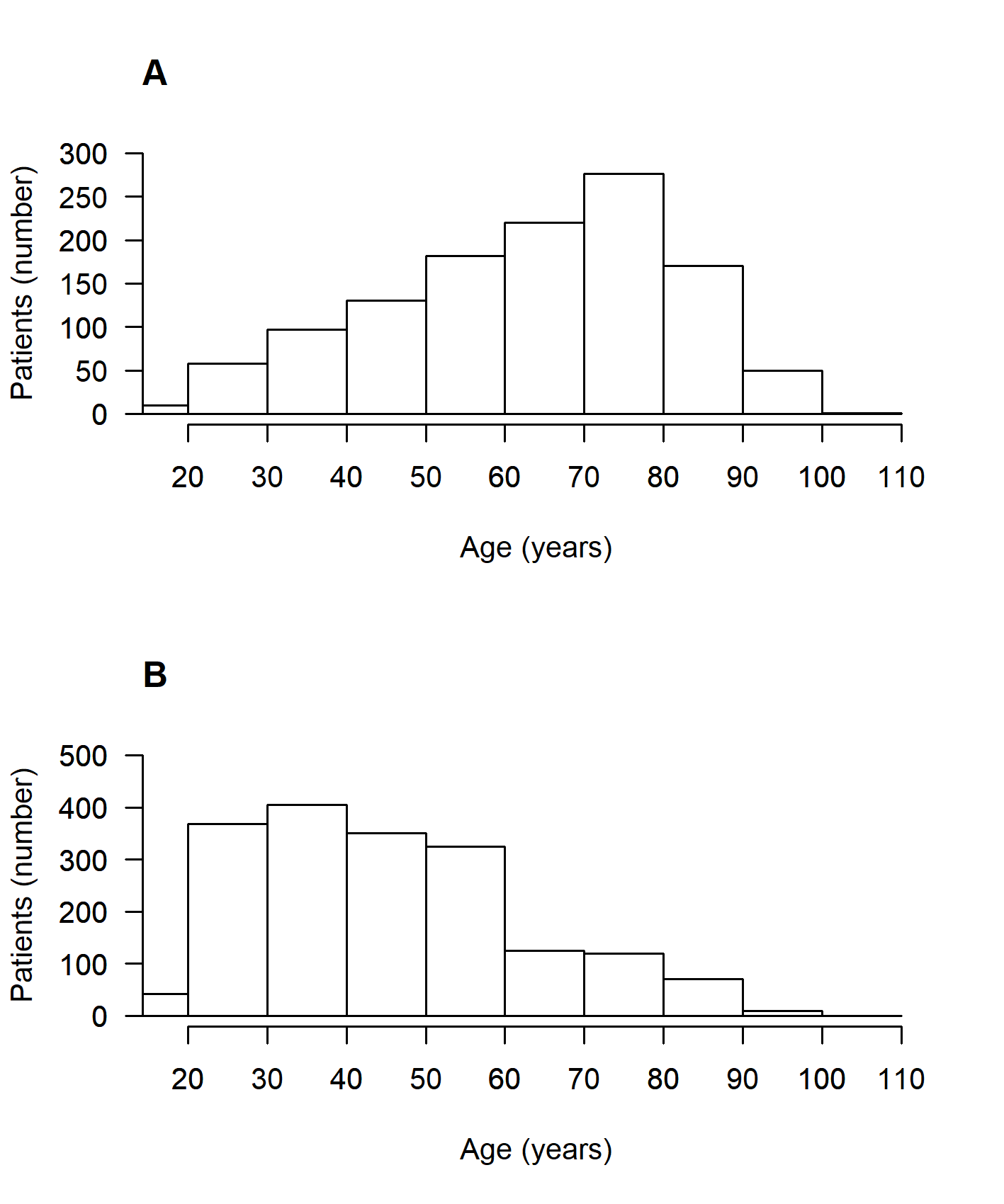

Supplement: S2 Fig — A. Inpatients. B. Outpatients. (TIFF) [file pone.0251661.s002.tiff]

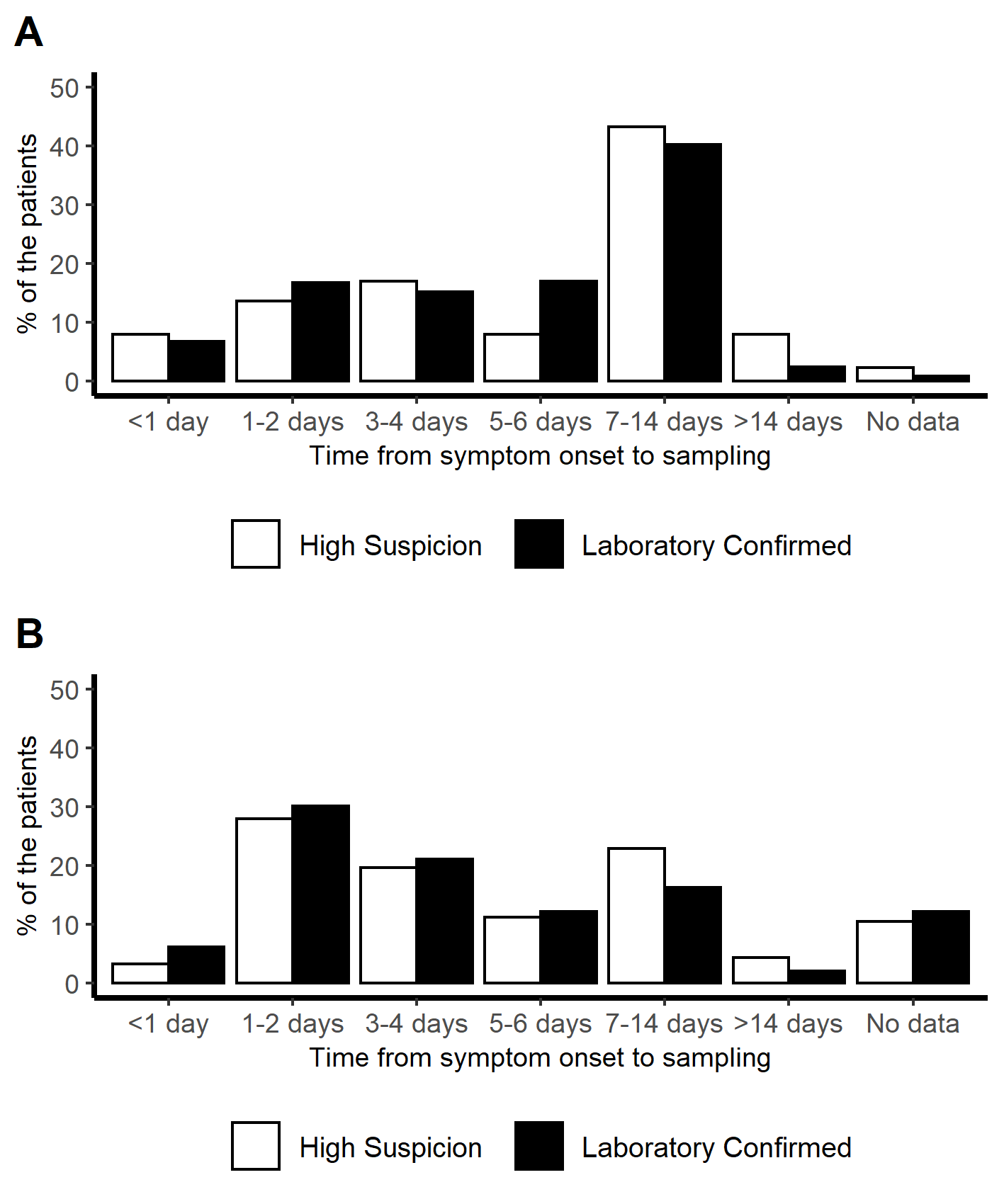

Supplement: S3 Fig — A. Inpatients B. Outpatients. (TIFF) [file pone.0251661.s003.tiff]

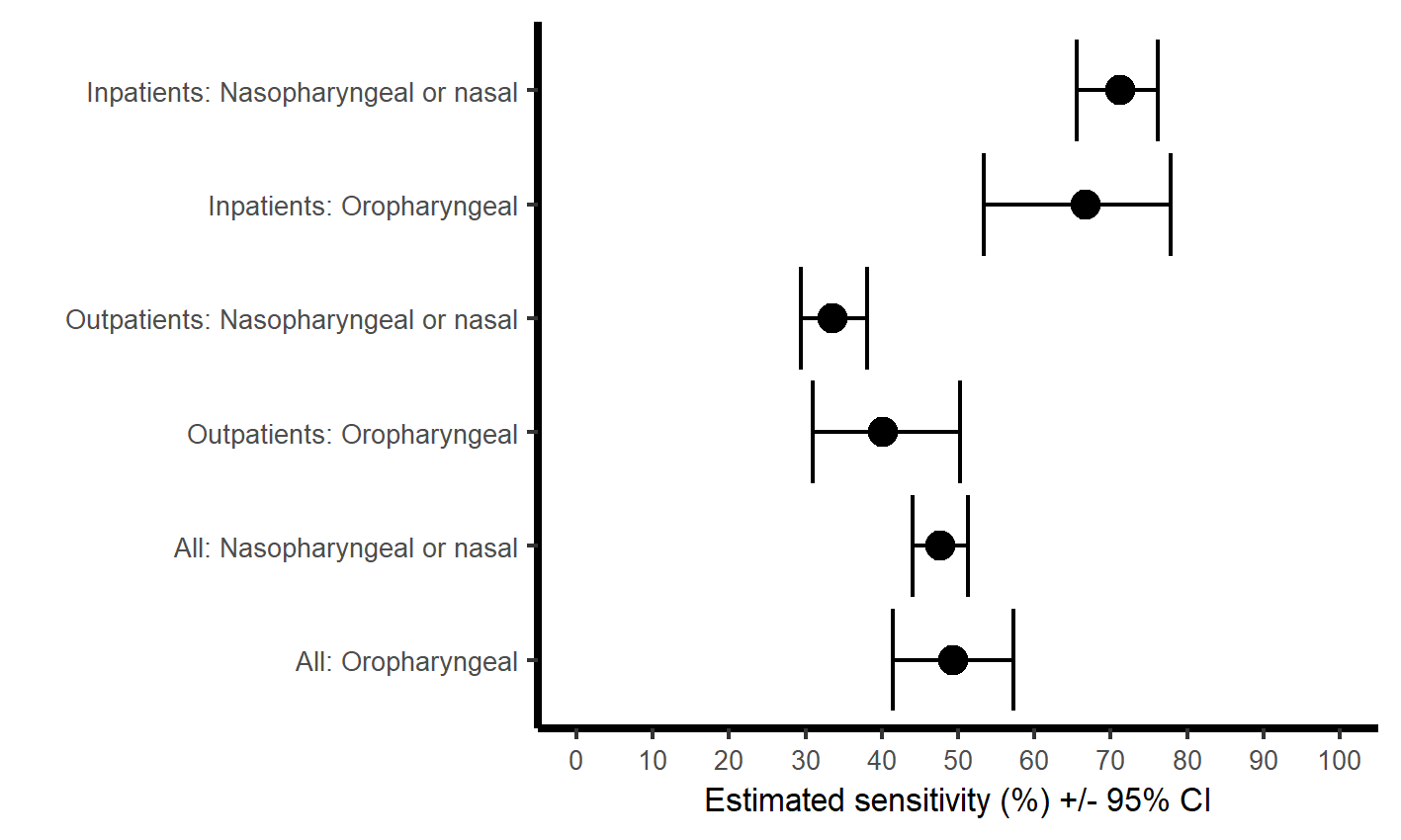

Supplement: S4 Fig — (TIFF) [file pone.0251661.s004.tiff]
