# Supplementary material for: Real-life clinical sensitivity of SARS-CoV-2 RT-PCR test in symptomatic patients
Source: PLoS One. 2021 May 21;16(5):e0251661. doi: 10.1371/journal.pone.0251661 (PMC8139477; doi:10.1371/journal.pone.0251661)
Supplement: S1 Table — (DOCX) [file pone.0251661.s005.docx]

**S1 Table**. List of SARS-CoV-2 RT-PCR testing criteria over the study period.

| Time period | SARS-CoV-2 testing criteria | Epidemic areas |
| --- | --- | --- |
| March 6^th^ - March 9^th^ 2020 | 1) Clinical picture of an acute respiratory infection: e.g. fever and/or cough and/or breathing difficulty and/or pneumonic imaging finding  AND 2) Possible exposure: travel to epidemic area or contact with laboratory confirmed  OR 3) Admission to ICU for respiratory infection, causative agent unknown | Mainland China, South Korea, Iran, Italy |
| March 9^th^ - March 15^th^ 2020 | Same as 6 March with an expanded epidemic area | Mainland China, South Korea, Iran, Italy, *Tyrol,* *Nordrhein-Westfalen (Germany)*^*^ |
| March 15^th^ - March 22^nd^ 2020 | 1) Quarantined individual experiencing symptoms of respiratory infection;  2) Admission to hospital for respiratory infection, causative agent unknown;  3) Individual over the age of 70 presenting with symptoms of respiratory infection;  4) Symptoms of respiratory infection AND possible exposure: travel or contact with lab. confirmed;  5) Nursing home resident with symptoms of respiratory infection AND suspicion of a respiratory infection epidemic in the facility | Italy, Tyrol  From 16 March onwards, all non-essential travel to and from Finland is prohibited |
| March 22^nd^ - April 13^th^ 2020 | Same as 15 March  and 6) health care personnel with symptoms of respiratory infection | No designated epidemic areas; all travel considered a risk |
| April 13^th^ 2020 onwards | 1) All individuals experiencing either respiratory or gastrointestinal symptoms;  2) Patients belonging to risk groups that are admitted to hospital for any reason |  |

*New areas in cursive
