# Supplementary material for: Real-life clinical sensitivity of SARS-CoV-2 RT-PCR test in symptomatic patients
Source: PLoS One. 2021 May 21;16(5):e0251661. doi: 10.1371/journal.pone.0251661 (PMC8139477; doi:10.1371/journal.pone.0251661)
Supplement: S2 Table — Inpatients were admitted to these COVID-19 cohort wards during the study period. Both laboratory confirmed and suspected COVID-19 patients were admitted to the cohort wards, except for two wards (COV_KNKINF and COV_KITEHO) to which only laboratory confirmed cases were admitted. (DOCX) [file pone.0251661.s006.docx]

**S2 Table.** List of COVID-19 cohort wards. Inpatients were admitted to these COVID-19 cohort wards during the study period. Both laboratory confirmed and suspected COVID-19 patients were admitted to the cohort wards, except for two wards (COV_KNKINF and COV_KITEHO) to which only laboratory confirmed cases were admitted.

| **Name of the ward** | **City** | **Hospital** | **Type of ward** | **Date when started as a cohort ward** |
| --- | --- | --- | --- | --- |
| MEKINFOS | Helsinki | Meilahti hospital area | ward | 4.3.2020 |
| MEKINFOSK4B | Helsinki | Meilahti hospital area | ward | 4.3.2020 |
| MEKKEU6A | Helsinki | Meilahti hospital area | ward | 16.3.2020 |
| MEKKEU6B | Helsinki | Meilahti hospital area | ward | 23.3.2020 |
| COV_MEKOS5 | Helsinki | Meilahti hospital area | ward | 1.4.2020 |
| MAOS5 | Helsinki | Malmi Hospital | ward | 13.3.2020 |
| PES4K | Vantaa | Peijas Hospital | ward | 30.3.2020 |
| JOKEU5 | Espoo | Jorvi Hospital | ward | 15.3.2020 |
| HYINFB-4 | Hyvinkää | Hyvinkää Hospital | ward | 8.4.2020 |
| RAUPPAVD | Raasepori | Raasepori Hospital | ward | 23.3.2020 |
| COV_KNKINF | Helsinki | Surgical Hospital | ward | 15.4.2020 |
| COV_KITEHO | Helsinki | Surgical Hospital | ICU | 9.4.2020 |
| MEM1 | Helsinki | Meilahti hospital area | ICU* | ** |
| PET | Vantaa | Peijas Hospital | ICU* | ** |
| PEPV | Vantaa | Peijas Hospital | ICU* | ** |
| JOU2 | Espoo | Jorvi Hospital | ICU* | ** |
| HYTEHVA | Hyvinkää | Hyvinkää Hospital | ICU* | ** |
| POPPKL | Porvoo | Porvoo Hospital | emergency department* | ** |

*Due to lack of patient lists of ICUs or one cohort ward at Porvoo Hospital, all inpatients with SARS-CoV-2 RT-PCR taken at ICUs or at the emergency department of Porvoo Hospital were included.

** All tests taken from 4 March to 15 April included.
