# Supplementary material for: Real-life clinical sensitivity of SARS-CoV-2 RT-PCR test in symptomatic patients
Source: PLoS One. 2021 May 21;16(5):e0251661. doi: 10.1371/journal.pone.0251661 (PMC8139477; doi:10.1371/journal.pone.0251661)
Supplement: S3 Table — (DOCX) [file pone.0251661.s007.docx]

**S3 Table**. Estimated SARS-CoV-2 RT-PCR sensitivity values in the laboratory confirmed and high suspicion group combined according to specimen type in the first SARS-CoV-2 RT-PCR test.

| **Specimen type*** | **Inpatients**  **Sensitivity (95 % CI)** | **Outpatients**  **Sensitivity (95 % CI)** | **All**  **Sensitivity (95 % CI)** |
| --- | --- | --- | --- |
| Nasopharyngeal | 195/274 | 154/458 | 349/732 |
|  | **71.2 %** | **33.6 %** | **47.7 %** |
|  | (65.5 – 76.2 %) | (29.4 – 38.1 %) | (44.1 – 51.3 %) |
| Oropharyngeal | 36/54 | 39/97 | 75/151 |
|  | **66.7 %** | **40.2 %** | **49.3 %** |
|  | (53.4 – 77.8 %) | (31.0 – 50.2 %) | (41.5 – 57.2 %) |
| Not known | 47/84 | 41/117 | 88/201 |
|  | **56.0 %** | **35.0 %** | **43.8 %** |
|  | (45.3 – 66.1 %) | (27.0 – 44.0 %) | (37.1 – 50.7 %) |

* Other sample types (Brocho-alveolar lavage, sputum, and tracheal aspirate) were excluded from analysis (n=4 for inpatients, n=2 for outpatients). Specimen type was unknown for 84 inpatients and 117 outpatients.
