# Supplementary material for: Real-life clinical sensitivity of SARS-CoV-2 RT-PCR test in symptomatic patients
Source: PLoS One. 2021 May 21;16(5):e0251661. doi: 10.1371/journal.pone.0251661 (PMC8139477; doi:10.1371/journal.pone.0251661)
Supplement: S4 Table — (DOCX) [file pone.0251661.s008.docx]

**S4 Table.** Estimated SARS-CoV-2 RT-PCR sensitivity values in the laboratory confirmed, and in the laboratory confirmed and high suspicion group combined according to delay (days) from symptom onset to first SARS-CoV-2 RT-PCR test.

| Delay (days) from symptom onset to sampling | **No of patients with first**  **RT-PCR positive** | **No of COVID-19 Laboratory confirmed patients** | **No of COVID-19 High suspicion patients** | **Total no of COVID-19 Laboratory confirmed and high suspicion patients** | **Sensitivity for COVID-19 Laboratory confirmed patients**  **% (95 % CI)** | **Sensitivity for COVID-19 Laboratory confirmed + High suspicion patients**  **% (95 % CI)** |
| --- | --- | --- | --- | --- | --- | --- |
| <1 day | 29 | 37 | 21 | 58 | 78.4 (62.8 - 88.6) | 50.0 (37.5 - 62.5) |
| 1-2 days | 118 | 129 | 132 | 261 | 91.5 (85.4 - 95.2) | 45.2 (39.3 - 51.3) |
| 3-4 days | 96 | 102 | 99 | 201 | 94.1 (87.8 - 97.3) | 47.8 (41.0 - 54.6) |
| 5-6 days | 77 | 88 | 55 | 143 | 87.5 (79.0 - 92.9) | 53.8 (45.7 - 61.8) |
| 7-14 days | 153 | 172 | 136 | 308 | 89.0 (83.4 - 92.8) | 49.7 (44.1 - 55.2) |
| >14 days | 10 | 13 | 26 | 39 | 76.9 (49.7 - 91.8) | 25.6 (14.6 - 41.1) |
| No data | 33 | 33 | 47 | 80 | 100 (89.6 - 100) | 41.2 (31.1 - 52.2) |
| Total | 516 | 574 | 516 | 1090 | 89.9 (87.2 - 92.1) | 47.3 (44.4 - 50.3) |
